# On the inclusion of self regulating branching processes in the working paradigm of evolutionary and population genetics

**DOI:** 10.3389/fgene.2013.00011

**Published:** 2013-02-19

**Authors:** Charles J. Mode, Candace K. Sleeman, Towfique Raj

**Affiliations:** ^1^Department of Mathematics, Drexel UniversityPhiladelphia, PA, USA; ^2^Nokia CorporationBurlington, MA, USA; ^3^Division of Genetics, Brigham and Women's HospitalBoston, MA, USA

**Keywords:** simulating evolution, mutations, density dependence, Monte Carlo methods, statistical summarizations, branching processes, embedded deterministic model

## Abstract

The principal goal of this methodological paper is to suggest to a general audience in the genetics community that the consideration of recent developments of self regulating branching processes may lead to the possibility of including this class of stochastic processes as part of working paradigm of evolutionary and population genetics. This class of branching processes is self regulating in the sense that an evolving population will grow only to a total population size that can be sustained by the environment. From the mathematical point of view the class processes under consideration belongs to a subfield of probability and statistics sometimes referred to as computational applied probability and stochastic processes. Computer intensive methods based on Monte Carlo simulation procedures have been used to empirically work out the predictions of a formulation by assigning numerical values to some point in the parameter space and computing replications of realizations of the process over thousands of generations of evolution. Statistical methods are then used on such samples of simulated data to produce informative summarizations of the data that provide insights into the evolutionary implications of computer experiments. Briefly, it is also possible to embed deterministic non-linear difference equations in the stochastic process by using a statistical procedure to estimate the sample functions of the process, which has interesting methodological implications as to whether stochastic or deterministic formulations may be applied separately or in combination in the study of evolution. It is recognized that the literature on population genetics contains a substantial number of papers in which Monte Carlo simulation methods have been used. But, this extensive literature is beyond the scope of this paper, which is focused on potential applications of self regulating branching processes in evolutionary and population genetics.

## 1. Introduction

Branching processes have been mentioned in books and papers dealing with population genetics ever since Fisher (1958), which is a revised version of a famous book on genetics and natural selection published in the late 1920s or early 1930s, introduced the idea as a framework for describing and analyzing the survival of a mutant gene. This theme was also explored by Li (1955) in a section of a book that extended Fisher's ideas in the sense that attention was focused on the survival of a genotype carrying a mutant gene rather than on single mutant gene considered by Fisher. Neither of these authors used the term, branching process, but they both realized that whether a mutant gene survives in a population depends on chance to a large degree. Simply put, if the individual carrying a mutant gene produces no offspring, or if the individual produces at least one offspring but his descendants fail to produce offspring in some generation, then the gene will eventually become extinct in a population with some probability. The number of offspring produced by any individual in a population is uncertain and to characterize this uncertainty mathematically the concept of an offspring distribution is essential component in formulating a branching process. In a paper published subsequent to the time his book on genetics and natural selection, Fisher (1939) dealt with the biological factors that affect the distribution of the number of offspring produced by an individual.

In books published on population genetics subsequent to the 1950s, the idea of formulating the survival of mutant genes in terms of a branching process was mentioned in subject indexes in some books, but not in others. Included in the books in which branching process are mentioned as models for the survival of mutant genes are Crow and Kimura (1970) and Ewens (2004). Among the books in which branching processes are not mentioned in the subject index in this connection are Cavalli-Sforza and Bodmer (1971), Hartl and Clark (1989), Buerger (2000), and Christiansen (2000). Indeed, many workers in population genetics that seem to hold the view that when population size is sufficiently large, there is no need for stochastic processes when formulating models of evolutionary dynamics with mutation, selection along with other forces that are thought to be driving evolution. There are, however, others working in the field whose methods belong to the stochastic paradigm, see, for example, the literature cited in the book Ewens (2004), where the Wright–Fisher process and related statistical models are the focus of attention.

Unlike population genetics, workers in probability and statistics have worked off and on with stochastic models that are now known as branching processes for over 100 years. For those readers who are interested in a brief history of this work, the historical prologue in Mode (1971) may be consulted along with the references cited therein. There is no need to go into this history in detail here, but, it is sufficient to mention that it was the problem of survival of well-known socially prominent names that prompted a variety of workers in several fields to analyze a stochastic model that later became known as the Galton–Watson branching process, (GW-process). Briefly stated, the fundamental problem for the GW-process was to find a formulation such that the probability that a famous name became extinct could be calculated.

Following the publication of the seminal book on branching processes by Harris (1963), a number of books on this subject were published in the English language by several authors. Among these books are Asmussen and Hering (1983), Athreya and Ney (1972), Jagers (1975), Kimmel and Axelrod (2002), and Mode (1971). A more recent book on branching processes and their applications in biology is that of Haccou et al. (2007). Among workers in applied probability, work on branching processes is an on-going research area, see, for example, the recent master's thesis (Alexander, 2010), and, as will be illustrated in the next section, in recent years this class of processes are also being applied increasingly in evolutionary and population genetics as we go forward into the twenty-first century.

For the most part, in all these books as well as in an extensive literature on branching process, the methods underlying a quest to formalize the predictions of a model rest in classical mathematical analysis and are most often expressed in terms of limit theorems as the number of generations or other time units become large. In particular, the book by Mode (1971) was devoted solely to multitype branching processes, which provided a framework for not only modeling the survival of mutant genes but also their emergence through the process of mutation, which was absent in the initial applications of branching processes by Fisher and others. With few exceptions, there is also a very serious limitation of most classical branching processes; namely, starting with an initial population of one or more individuals, the population either becomes extinct or its growth is unbounded, which has been rightfully criticized by biologists and other workers in various fields of science.

Although only a few textbooks published up to and including the year 2000 had covered branching processes, in recent years papers applying various classes of branching processes in population genetics and related fields have been published in various journals. An example of such a paper is that of Alsmeyer et al. (2011) in which the limiting genotypic growth rates and limiting genotypic frequencies of Y-linked genes are studied in a two-sex monogamous population. In another paper, Iwasa et al. (2003) applied a class of branching processes based on Markov jump processes in continuous time to study the evolutionary dynamics of escape from biomedical intervention due to mutations in the genome of a disease causing organism such as HIV. The working paradigm underlying these papers differed significantly. In the paper of Alsmeyer et al. (2011), which was rooted in the extension of classical Galton–Watson process, and is an extension of this class of branching processes. In the paper by Iwasa et al. (2003), however, attention was centered on the infinitesimal generator of a multitype Markov jump process in continuous time. On the other hand, the paper by Lehe et al. (2012) on the rate of a beneficial mutations surfing on the wave of a range expansion is based on a pioneering paper of R. A. Fisher and seems to be related to a class of branching diffusion processes. Another paper on the applications of branching processes is that of Patwa and Wahl (2010), who applied these processes to estimate the substitution rate at which beneficial mutations occur and fix in populations of lytic viruses whose growth is controlled by periodic population bottlenecks. A multitype Galton–Watson process was applied directly by Serra and Haccou (2007) to study the dynamics of escape mutants, when the expected number of offspring by mutant genotypes is so small that they are doomed to extinction. In a different setting, Wild (2011) used a multitype Galton–Watson process to approximate a complex Markov chain in discrete time to compute an inclusive fitness parameter, which was the Perron–Frobenius root of a regular expectation matrix of non-negative elements that plays a fundamental role in this class of multi-type processes.

The class of self regulating branching processes under consideration in this paper is fundamentally different from those mentioned above in that the probability of survival of an individual in any generation to produce the offspring of the next generation depends on the total population size of that generation. Furthermore, unlike the methods used in the papers cited above, in which various analytic ideas from classical mathematics were used to work out the predictions of some branching process formulation, in the class of branching processes under consideration computer intensive methods play a fundament role in working out the predictions based on a self regulating branching process formulation. Thanks to the development of powerful desk, lap top computers and networks of computers that are available to either individuals or teams of researches, it is now possible to formulate and analyze, by empirical computer experiments, the evolutionary predictions of multitype self regulating branching processes, which have the property that a population evolving from one or more initial individuals either becomes extinct or converges in distribution to a state such that total population size is bounded because of environmental constraints. Due to the development of Monte Carlo simulation methods in a number of fields of science, the applications of these methods make it possible to work out some of the evolutionary predictions of a branching process formulation that may not be easily attainable using classical mathematical analysis. Moreover, in principle, Monte Carlo simulation methods are based on intuitive notions that render them useful to scientists working in a laboratory, provided that software is available to suit their needs.

The use of Monte Carlo simulation methods also results in insights that, in general, at not attainable when the predictions of a formulation are worked out by using limit theorems. For even though the results of limit theorems may be very interesting from an evolutionary point of view, the methods used in proving these theorems are usually not informative as to the evolutionary time taken for a population to converge to these limits. But, if an investigator uses Monte Carlo simulation methods to compute a sample of realizations of stochastic process over some evolution time period involving thousands of generations and summarizes the simulated data using statistical methods, then it is possible to gain insights into the time taken to converge to limits, which may be a point or set of points or even some form of a stationary distribution.

There is another aspect of the class of self regulating branching processes under consideration that differentiates them from those described in the books and papers cited above. By using statistical notions to estimate random variables or functions, it is possible to development systematic procedures for embedding multivariate non-linear difference equations in a stochastic process, whose sample functions take values in an infinite set of multi-dimensional vectors of non-negative integers. These non-linear equations have the same parameter space as the process so that, given assigned numerical values of some point in the parameter space, it is possible to conduct a computer experiment such that not only a sample of Monte Carlo realizations are computed but also a numerical solution of the embedded non-linear difference equations. By comparing the predictive trajectories of the embedded deterministic model with the trajectories of the stochastic process, based on a sample of Monte Carlo realizations of the process, it is possible to empirically analyze the extent to which deterministic predictions are consistent or not consistent with those of the process.

There is also an interesting theoretical property of the embedded non-linear difference equations that is worthy of mention. It can be shown by examples, that at some points in the parameter space, the non-linear difference equations become chaotic. It is also possible to compute a sample on Monte Carlo realizations of the process based in this point in the parameter space so that deterministic predictions may be compared with those of the process. Such comparisons also have interesting implications for the development of new methods of statistical inference. These interesting prospects for future research on self regulating branching process will not be pursued in this paper, but it is hoped that some probabilistic analysts will join in the quest to work out further predictions based on formulations of self regulating branching processes.

Briefly, in order to make this paper self contained, the next section is devoted to an illustrative formulation of a one type self regulating branching process followed a section in which the type one case is extended to the case of a multitype self regulating branching process. The next section is devoted to an overview of the contents of a book by to two leading authors of this paper on stochastic processes in genetics and evolution that, among other topics, contains reports of computer simulation experiments on applying self regulating branching process in the study of evolving populations in which the forces of mutation and selection are studied empirically. In the last section on the paper, the results of a computer simulation experiment are reported in which the predictions of the embedded deterministic model differ significantly from those of statistically summarized sample simulated data generated by Monte Carlo simulation methods.

## 2. One type self regulating branching processes

For the sake of simplicity, all offspring distributions that will be used in this review paper will be one parameter Poisson distributions. Let λ denote a positive number and again let *N* denote a random variable taking values in the set of non-negative integers I={n∣0, 1, 2, …}, then it will be assumed that the offspring distribution has the form:

(2.1) $$P[N=n]=f(n)=e^{-\lambda }\frac{\lambda^{n}}{n!}$$

for all n∈I. It is well-known that the expectation of *N* is *E*[*N*] = λ so that λ may be interpreted as the mean or expected number of offspring contributed to the next generation for any individual in an evolving population. As is also well-known, the variance of the random variable *N* for the Poisson case is var[*N*] = λ. It can be shown that *m* = λ is a critical parameter in determining the probability that a population evolving from an initial population of one or more individuals becomes extinct or grows without bound. If a reader is interested in the details underlying this statement it is suggested that the Book Harris (1963) be consulted.

Let *X*(0) = *x*_0_ denote the number of individuals in the initial population and let *X*(*t*) for *t* = 1, 2,… denote the random number of individuals in the population in generation *t* that are descendants of the individuals in the initial population. If *x*_0_ = 1, then *X*(1) is a realization of the random variable *N* with a Poisson distribution. But, if *x*_0_ > 1, then, by assumption, let *N*_*k*_ for *k* = 1, 2,…, *x*_0_ be independent and identically distributed random variables whose common distribution is that of *N*. Then,

(2.2) $$X(1)=\sum_{k\;=\;1}^{x_{0}}N_{k}.$$

In general, suppose that in generation *t*, the number of individuals in the population is given by the random variable *X*(*t*), and given *X*(*t*) ≥ 1, let *N*_*k*_ for *k* = 1, 2,…, *X*(*t*) denote a conditionally independent and identically distributed random variables whose common distribution is that of the random variable *N*. Then, the number of individuals in generation *t* + 1 is given by the random sum:

(2.3) $$X(t+1)=\sum_{k=1}^{X(t)}N_{k}$$

and *X*(*t* + 1) = 0 if *X*(*t*) = 0. The simple branching process just defined is also known as the GW-process, the Galton–Watson process.

From the definition of the GW-process, it is easy to see how one could compute Monte Carlo realizations of the process. For all ones needs to do is to call realizations of Poisson random variable as indicated by the formulas displayed above. Many software packages often contain procedures to simulate realizations of Poisson random variables, given an assigned numerical value of the parameter λ, but if such a package is not available to an investigator, it is a straight forward task to write such a program in a computer programming language of one's choice by consulting existing literature on algorithms for simulating realizations of random variables with a specified distributions. More details on this subject will be given in a subsequent section.

Let η(*t*) = *E*[*X*(*t*)] be the unconditional expectation of the random variable *X*(*t*), denoting the number of individuals in the population in generation *t*. Then, from the above equation, it can be seen that the conditional expectation of *X*(*t* + 1), given *X*(*t*), is

(2.4) E[X(t+t)∣X(t)]=X(t)λ,

because *E* [*N*_*k*_] =λ for all *k* = 1, 2,…, *X*(*t*). By taking unconditional expectations, it can be seen that,

(2.5) η(t+1)=E[E[X(t+t)∣X(t)]]=E(X(t))λ=η(t)λ.

If this equation is iterated, then it can be shown that,

(2.6) $$\eta (t)=x_{0}\lambda^{t}.$$

Thus, if λ < 1, then η (*t*) → 0 as *t* ↑ ∞. But, if λ = 1, then η (*t*) = *x*_0_ for all *t* ≥ 1, and if λ > 1, then η (*t*) ↑ ∞ as *t* ↑ ∞, indicating that the growth of the population is unbounded. To correct this serious flaw in the GW-process, the concept of a self regulating branching process will be introduced.

Because in a self regulating branching process all offspring produced in a given generation may not survive to produce offspring in the next generation, the fundamental equation (2.3) characterizing a GW-process needs to be modified. Let the random variable *Y* (*t*) denote the random number of offspring produced by all individuals in generation *t*. Then, from the fundamental equation (2.3), it follows that,

(2.7) $$Y(t)=\sum_{k=1}^{X(t)}N_{k}.$$

By assumption, not all the offspring represented by the random variable *Y*(*t*) will survive to produce offspring in generation *t* + 1. It becomes necessary, therefore, to introduce a survival probability to stochastically characterize the survival of offspring in any generation *t* as a function of population size *X*(*t*).

A survival function that is often applied in applied probability is the well-known Weibull, which has the parametric form:

(2.8) $$S(t)=\exp[-{\left(\beta t\right)}^{\alpha }],$$

where α and β are positive parameters. The usual interpretation of this function is based on the following considerations. Suppose at some time 0 a live individual that may subsequently die is observed. Then, *S* (*t*) is interpreted as the probability that this individual is still alive at time *t*. In the context of a one type self regulating branching process, this survival function needs to be modified to accommodate the size of the population in any generation *t*. Let *S* (*t* | *X* (*t*)) denote the conditional probability that any offspring produced in generation *t* survives to produce offspring in generation *t* + 1. Then, this conditional probability will be chosen as:

(2.9) $$S(t|X(t))=\exp[-{\left(\beta X(t)\right)}^{\alpha }].$$

Intuitively, if population size in any generation exceeds the carrying capacity of the environment, then, due to the competition for resources, it is less likely that an offspring produced in any generation *t* will survive to produce offspring in generation *t* + 1. Observe that if β*X* (*t*) ≤ 1, an offspring produced in generation *t* is more likely to survive to produce offspring than if β*X* (*t*) > 1. In particular, if β = 0, then all offspring produced in any generation survive with probability one to produce offspring in the next generation so that in this special case a self regulating branching process reduces to the classical GW-process. From a conceptual point of view, it is useful to think of the parameter β as the inverse of the carrying capacity of the environment. Thus, for example, if one supposes that the carrying capacity of the environment is 10^6^, then β would be chosen as β = 10^−6^. Observe that whenever *X* (*t*) ≥ 10^6^, it is less probable that an offspring in generation *t* will survive to produce offspring in generation *t* + 1. It is of interest to observe that if the parameter α = 1, then the survival function under consideration reduces to that of the exponential distribution.

Having completed the definition of the conditional probability that an offspring produced in generation *t* survives to produce offspring in generation *t* + 1, it is now possible to complete the formulation of a one type self regulating branching process. By assumption, it will be supposed that *X* (*t* + 1), the random number of individuals who may produce offspring in generation *t* + 1, has a binomial distribution with sample size or index *Y* (*t*) and conditional probability *S* (*t* | *X* (*t*)), given population size *X* (*t*) in generation *t*. Thus, to compute a realization of the random variable *X* (*t* + 1), one needs to have a program to compute realizations of binomial random variables. Such programs are often contained of many software packages, particularly those designed for applications arising in the probability and statistics community as well as the communities of users of these methods.

At this point in the exploration of ideas underlying self regulating branching processes, it is appropriate to outline the idea of embedding a deterministic model in a stochastic process. Although the technical details will be omitted, it can be shown that,

(2.10) E[X(t+1)∣X(t)]=X(t)S(t∣X(t))λ

is the correct formula for the conditional expectation of *X* (*t* + 1), given *X* (*t*), for every generation *t* = 0, 1, 2,…. From the point of view of estimating a value of the sample function of a process at time *t* + 1, it is well-known that *E* [*X* (*t* + 1) | *X* (*t*)] is the function that is the best fit in the sense that the expected mean square error of deviations from the random function *X* (*t* + 1) is a minimum.

Unlike the case of the simple GW-process, it is not possible to derive a useful unconditional expectation of the random function *X* (*t* + 1), because the survival probability *S* (*t* | *X* (*t*)) is a non-linear function of the random function of *X* (*t*), see formula (2.9). But, the value *X* (0) is known, because this value must be assigned by the experimenter as a positive integer. Thus,

(2.11) $$\hat{X}(1)=E[X(1)|X(0)]=X(0)\;S\left(t|X(0)\right)\lambda$$

is the best estimator of the random function *X* (1) in the sense of minimum mean square error. Given this formula for $\hat{X}\left(1\right)$ from Equation (2.10), it can be seen from Equation (2.10) that

(2.12) $$\hat{X}(2)=\hat{X}(1)\;S(t|\hat{X}(1))\lambda$$

is a reasonable estimator of the random function *X* (2), even though, in general, it may not be optimal in the sense of minimum mean square error. By continuing this line of thought, we arrive at the non-linear recursive system:

(2.13) $$\hat{X}(t+1)=\hat{X}(t)\;S(t|\hat{X}(t))\lambda ,$$

for *t* = 0, 1, 2,…, which, by definition, is a non-linear deterministic model embedded a stochastic process called a one type self regulating branching process. Such a name is justifiable in the sense that the eventual size an evolving population may attain is regulated by the carrying capacity of the environment.

The parameter space of both the process and the embedded deterministic model is the set:
(2.14)
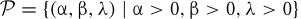

of triplets of positive real numbers, and for each point 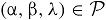. Given assigned numbers parameters by an experimenter, it possible to simulate a sample of trajectories, sample functions, of the process. Moreover, when this sample of trajectories is summarized statistically, it can be compared with the one trajectory computed by using the embedded deterministic at this point in the parameter space. By using graphical and other methods for an informative display of data, real or simulated, it is possible to assess experimentally how well the embedded deterministic model predicts the behavior of the process in the sense that the deterministic model may be a measure of central tendency for the variable sample functions of the process.

When viewing the deterministic model from a purely mathematical perspective, it is helpful to write the function of the right in Equation (2.13) in the explicit form:

(2.15) $$h(x)=x\exp[-{\left(\beta x\right)}^{\alpha }]\lambda$$

with a view of making connections with well-known deterministic mathematical properties of trajectories based on the iteration of non-linear functions such as that in Equation (2.15). That basically any trajectory of the embedded deterministic model, given assigned value of a parameter point (α, β, γ), is an iteration of the function in Equation (2.15) easy to see. For if an experimenter chooses an initial positive integer *x*_0_, then by computing the sequence *x*_1_ = *h* (*x*_0_), *x*_2_ = *h* (*x*_1_) = *h*(*h*(*x*_0_)),… and so on it can be seen that this sequence is realized trajectory of the embedded deterministic model. Over time periods of decades, mathematicians dealing with non-linear deterministic dynamic models have worked out the properties of iterated continuous functions, and the knowledge so gained is an important starting point when attempting to work out ideas centering around the idea: in what sense may an embedded deterministic model be used to predict the behavior of the sample functions of the stochastic process.

There is an extensive literature dealing with chaos in non-linear systems of deterministic equations as dynamic models of various phenomena. For those readers who prefer to learn about these systems through a combination of lectures and readings, it is suggested the recent lectures of Devaney (2011) as well as the accompanying notes be consulted. In these lectures and notes, a famous theorem by Sharkovsky, which was published in Russian in the 1960s, is discussed. The technical details will be omitted, but it is based on a special ordering of a set of positive integers called the Sharkovsky ordering, see Devaney (2011) for details. A non-technical version of the theorem may be stated as follows. Suppose a function *h* (*x*) on the real number line is continuous. Then, if the trajectory of this function has a period of a prime number *n*, then it must have a prime period *k* for any integer that follows *n* in the Sharkovsky ordering. If one types the title, Sharkovsky's theorem, into an internet search engine, more detailed information on this theorem may be obtained.

Consider, for example, the function defined in Equation (2.15). By definition, if we let *h* (*x*) = 0 if *x* < 0, then *h* (*x*) is continuous on the real number line ( −∞, ∞) at every parameter point 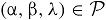. An incomplete analysis of the model under consideration may be found in chapter 9 of Mode and Sleeman (2012). A fixed point of the function *h* (*x*) is a point *x*_*f*_ such that *x*_*f*_ = *h* (*x*_*f*_). For the model under consideration, it is possible to derive a formula for the fixed point *x*_*f*_ for every point 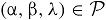 in the parameter space. Moreover, a condition was derived such that, at some set in the parameter space, a trajectory of the deterministic model will always be attracted to the fixed point, i.e., it is stable. At other points in the parameter space, a trajectory is repelled from the fixed point so that it is unstable. Furthermore, in exploratory numerical experiments, it has been observed that some trajectories of the embedded deterministic model had periods of two or greater at some points in the parameter space. Generally speaking, such periods were observed when the parameter λ was relatively large, indicating that on average each individual contributed a rather large number of offspring to the next generation. It is known that periodic systems exist in chemistry, but it is not clear whether such systems exist in biology and, in particular, population genetics.

At some of the points in the parameter space in which it was observed that the trajectory of the embedded deterministic model was periodic, a sample of Monte Carlo realizations of the process was also computed at such a parameter point. In general, when the simulated realizations of the process were summarized statistically, the periodicity of the embedded deterministic model was somewhat blurred in the estimated quantiles of the process. For example, the median quantile *Q*50 showed evidence suggesting periodicity, but in the simulated data was much less clear than in the embedded deterministic trajectory. For readers who may be interested in more details on these experiments, it is suggested that chapter 9 in Mode and Sleeman (2012) be consulted.

At this point, it should be noted that the choice of a Poisson distribution for the offspring distribution was a conservative one in the sense that samples from a Poisson distribution are well behaved, because the moments of this distribution are all finite. If one were to chose an offspring distribution such that the mean or expected value was finite but some of the higher order moments were infinite, then the sample function of the process would be more variable, which suggests that the predictions based on the embedded deterministic model may be less useful, because the sample functions of the process may vary among realizations of the process more than those for a Poisson offspring distribution. When the emergence and survival on new mutations in a population may be a seminal event in the evolution of a population is the focus of attention, a one type self regulating branching process is not a useful model, because the possibility of new mutations is not accommodated in this model. This relatively simple model does, however, provide a useful window into what to expect when multitype self regulating process are introduced as a framework for studying the emergence and survival of new mutations in an evolving population as will be shown in the next section.

## 3. Multitype self regulating branching processes

In the context of genetics and evolution, the idea of type in a multitype branching process will correspond to a genotype. For the sake of simplicity, attention will be focused on the case of three genotypes, and initially it will be assumed that are two forces driving the evolution of a population, reproductive success and mutations among the three genotypes. Let G denote the set of three genotypes that will be symbolized by ν = 1, 2, 3. To characterize reproductive success stochastically, let *N*_ν_, such that ν∈G, denote a set of random variables taking values in the set I of non-negative integers, and suppose, as in section 2, that the density function for each of these random variable is a Poisson with parameter λ_ν_ for all ν∈G. Then a useful measure of reproductive success for each genotype is the expectation *E* [*N*_ν_] = λ_ν_ for every ν∈G. If the value of any one of these parameters exceeds the others, then the genotype corresponding to this value would have a selective advantage over the others, in the sense that in every generation individuals of this genotype would, on average, contribute more offspring to the next generation.

By assumption, the other force driving evolution of the population is mutation among the three genotypes. Let μ_*ij*_ denote the conditional probability that parental individual of genotype i∈G produces an offspring of genotype j∈G per generation. It is often the case that in classical population genetics the idea of rates of mutations are used rather than probabilities of mutation. But, in the classical case, rates are often defined as follows. Suppose some large number of parents produce a large number of offspring *k* in some generation, and among these offspring, it is observed that *k*_0_ are of the some mutant type. Then, by definition, the rate of mutation is the fraction *k*_0_/*k*. But, this definition is precisely that used to define a probability of an event within the frequency interpretation of probability. Hence, to be consistent with terminology that is widely used terminology in probabilistic formulations, the word probability will be used when referring to the uncertainty with which mutations occur.

For a more complete interpretation of these conditional probabilities, it is useful to represent them in the matrix form:

(3.1) $$M=\left(\begin{array}{ccc} \mu_{11} & \mu_{12} & \mu_{13}\\ \mu_{21} & \mu_{22} & \mu_{23}\\ \mu_{31} & \mu_{32} & \mu_{33} \end{array}\right).$$

In this matrix, μ_11_, for example, is the conditional probability that an individual of genotype 1 produces an offspring of genotype 1, but μ_12_ and μ_13_ are, respectively, the conditional probabilities that an individual of genotype 1 produces an offspring of genotype 2 or 3. For every i∈G,

(3.2) $$\sum_{j\;\in G}\mu_{ij}=1$$

so that each row of the matrix M may be thought of as the vector of probabilities for a three dimensional multinomial distribution. Let the row vector ***p***_i_ = (μ_*i*1_, μ_*i*2_, μ_*i*3_) denote row i∈G of the matrix M in Equation (3.1). Because of condition (3.2), it can be seen that one is free to choose six parameters of the model regarding mutations. Then, given these choices, the constraints imposed by Equation (3.2) will determine the other three parameters in the matrix in Equation (3.1) of conditional mutation probabilities.

In an evolving population, let the random function *X*_*i*_ (*t*), taking values in the set I of non-negative integers, denote the number of individuals of genotype i∈G in the population in generation *t*. To initiate a Monte Carlo simulation experiment, it will be necessary to assign values to each of these random functions in generation *t* = 0. Let *X*_*i*_ (0) = *x*_*i*_ (0) for *i* = 1, 2, 3 denote the initial number assigned for each of the three genotypes. Suppose in generation *t* = 0, 1, 2,… there are *X*_*i*_ (*t*) ≥ 1 individuals of genotype *i*, and given *X*_*i*_ (*t*), let *N*^(*k*)^_*i*_ for *k* = 1, 2,…, *X*_*i*_ (*t*) denote a set of conditionally independent random variables whose common distribution is that of the Poisson random variable *N*_*i*_ with expectation λ_*i*_ > 0. For a fixed *k*, let ***Z***^(*k*)^_*i*_ = (*Z*^(*k*)^_*ij*_ | *j* = 1, 2, 3) denote a random three dimensional vector whose components *Z*^(*k*)^_*ij*_, for *j* = 1, 2, 3, are the random numbers of offspring of genotype *j* produced by the individual *k* of genotype *i* in generation *t*. Then for each *k* = 1, 2,…, *X*_*i*_ (*t*), the random vector ***Z***^(*k*)^_*i*_ has a multinomial distribution with index *N*^(*k*)^_*i*_, sample size, and probability vector ***p***_i_ = (μ_*i*1_, μ_*i*2_, μ_*i*3_).

Let the random function *Y*_*ij*_ (*t*) denote the number of offspring of genotype *j* = 1, 2, 3 produced by the *X*_*i*_ (*t*) of genotypes *i* in generation *t*. Then, for *X*_*i*_ (*t*) ≥ 1, *Y*_*ij*_ (*t*) is the random sum:

(3.3) $$Y_{ij}(t)=\sum_{k=1}^{X_{i}(t)}Z_{ij}^{\left(k\right)},$$

and if *X*_*i*_ (*t*) = 0, then *Y*_*ij*_ (*t*) = 0. Let the random function *Y*_*j*_ (*t*) denote the total number of offspring of genotype *j* produced by all of the three genotypes in generation *t*. Then, under the assumption that mutations among all genotypes may occur, it follows that,

(3.4) $$Y_{j}(t)=\sum_{i=1}^{3}Y_{ij}(t)=\sum_{i=1}^{3}\sum_{k=1}^{X_{i}(t)}Z_{ij}^{\left(k\right)}$$

for *j* = 1, 2, 3.

In any Monte Carlo simulation experiment based on the above outline of random functions describing the evolution of a multitype branching process, two steps will be required. The first step is to compute Monte Carlo realizations of the Poison random variables *N*^(*k*)^_*i*_ *k* = 1, 2,…, *X*_*i*_ (*t*) for *i* = 1, 2, 3. Then, for a particular *k*, one needs to simulate a realization of the multinomial random vector ***Z***^(*k*)^_*i*_ for *i* = 1, 2, 3. To execute such a simulation, it is suggested that a reader consult (Mode and Gallop, 2008), where an algorithm for computing realizations of random vectors following a multinomial distribution with index *N* and probability vector ***p*** was described in detail. It should also be mentioned that the computational efficiency of computing realizations of many Poisson random variables may be significantly increased by using a Central Limit Theorem approximation. An interested reader may consult Mode and Sleeman (2012) for details, but we will not go into the details here.

Having outlined a set of algorithms for simulating the total number of offspring of each genotype produced in any generation, the next step in the formulation of a self regulating multitype branching process is to introduce survival probabilities for each of the three genotypes. In any generation *t*, let the random function

(3.5) $$T(t)=\sum_{i=1}^{3}X_{i}(t)$$

denote total population size in generation *t* for *t* = 0, 1, 2,…. Then, just as for the one type branching process described in section 1, the conditional survival function for an offspring of genotype *i* to survive and reproduce in generation *t* + 1 is, by definition,

(3.6) $$S_{i}(t|T(t))=\exp[-{\left(\beta_{i}T(t)\right)}^{\alpha_{i}}]$$

for *i* = 1, 2, 3, where the parameters satisfy the conditions β_*i*_ > 0 and α_*i*_ > 0 for all genotypes i∈G.

At this juncture, it should be noted that the assignment of values for six parameters in the three survival functions in Equation (3.6) will be required in any simulation experiment, which raises the dimension of the parameter space of the model to 15. It becomes helpful, therefore, to reduce the dimension of the parameter space by assigning fixed values to selected parameters. For the survival functions in Equation (3.6), the beta parameters, which reflect the carrying capacity of the environment, are more critical for the execution of computer simulation experiments than the alpha parameters. Consequently, in all the computer experiments reported in this paper, the alpha parameters will be assigned the value α_*i*_ = 2 for *i* = 1, 2, 3 so that the dimension of the space of the model reduces to 12. It should be mentioned, however, that an experimenter would be free to assign any parameter values of his choosing.

At first sight, a parameter space of 12 dimensions may be difficult for an experimenter of cope with. But, on the other hand, it also provides a framework to study the effects of more than one component of natural selection in any Monte Carlo simulation experiment. For example, given an assignment of values for the conditional mutation probabilities, an experimenter would be free to choose different values for lambda parameters among the three genotypes that quantify differential reproductive success by genotype, which would be one component of natural selection. By choosing different values of the beta parameters in the survival function for each genotype, differential capacities to compete for resources among the three genotypes could also be studied as a component of natural selection in computer simulation experiments. In a subsequent section, the results of a computer experiment will be reported to illustrate how the two components of natural selection just mentioned can be studied in various combinations of components or for each component separately in Monte Carlo simulation experiments.

The last step in the formulation of a multitype self regulating branching process is to define a procedure for simulating realization of the random functions *X*_*i*_ (*t* + 1) denoting the number of individuals of genotype *i* = 1, 2, 3 in the population in generation *t* + 1. As above, let the random function *Y*_*i*_ (*t*) denoted the number of offspring of genotype *i* = 1, 2, 3 produced in generation *t*. Then, for each *i* = 1, 2, 3, *X*_*i*_ (*t* + 1) is a realization of a binomial random variable with index *Y*_*i*_ (*t*) and conditional probability *S*_*i*_ (*t* | *T* (*t*)).

Having outlined the fundamentals underlying a stochastic multitype self regulating branching process with three genotypes, the stage has been set for a description of the embedded deterministic model. Let ***M***(***X*** (*t*)) denote a 3 × 3 random matrix of conditional expectations of the number of offspring produced by individual of genotype *i* for *i* = 1, 2, 3 in any generation *t*. Then, the vector

(3.7) $$S_{i}(t|T(t))\lambda_{i}(\mu_{i1},\mu_{i2},\mu_{i3})$$

for *i* = 1, 2, 3 is row *i* of the matrix ***M***(***X*** (*t*)). Let the 1 × 3 vector

(3.8) $$\hat{X}(t)=\left({\hat{X}}_{1}(t),{\hat{X}}_{2}(t),{\hat{X}}_{3}(t)\right)$$

denote the estimate of the 1 × 3 random population vector

(3.9) $$X(t)=\left(X_{1}(t),X_{2}(t),X_{3}(t)\right)$$

in generation *t* = 0, 1, 2,…. Then,

(3.10) $$\hat{X}(t+1)=\hat{X}(t)M\left(\hat{X}(t)\right)$$

for *t* = 0, 1, 2,….

In the vector valued function on the right in Equation (3.10) is non-linear in each of its components. It has been shown in computer experiments reported in chapter 10 of Mode and Sleeman (2012) that at some points in the parameter space the vector valued iterates of the function on the right in Equation (3.10) become irregular or chaotic, but at other points in the parameter space the iterates of this function behave a more regular fashion and converge to a limit determined by the carrying capacity of the environment. Generally speaking, chaotic trajectories usually arise when the lambda parameters are large, indicating that on average each individual contributes a large number of offspring to the next generation.

A question arises is: for what biological systems will the model under consideration be applicable? One could write a short essay in attempting to answer this question, but only a few brief remarks will be made here. In principle, any single or multicellular species that produces asexually could be a candidate for applications of the model under consideration. Included among these species are bacteria that reproduce by binary cell division. Furthermore, even some species of chemical molecules that can copy itself but at times there may be errors in the copying process, which would provide variation for the process of Darwinian evolution to occur, would also be candidates for applications of the model under consideration. For a set of interesting lectures on the origin of life, the lectures of Hazen (2005) may be consulted.

## 4. Overview of book's contents

In this section, the word “book” will refer to the Mode and Sleeman (2012), which contains 15 chapters. It should be made clear at the outset, that none of the references that are listed at the end of each chapter will be cited in this paper with a only a few exceptions. For some chapters the list of reference is too long to cite here. Moreover, those references that were cited in the book pertain only to the topics considered in the book. While writing the book, the decision to restrict the number of references cited was made, because any attempt to provide and overview of the large and growing literature on statistical methods designed for applications in molecular biology and genetics as well as books and papers in journals devoted to Bioinformatics was beyond the scope of the book. But whenever it is deemed appropriate, the contents of the cited references will be briefly stated.

Chapter 1 is devoted to an introduction of mathematical probability and its applications to Mendelian genetics. Also contained in this chapter is the derivation of a number of well-known distributions that are subsequently used throughout the remainder of the book. Chapter 2 contains a theoretical account linkage and recombination at multiple loci in which recombination probabilities are defined and derived for any number *N* ≥ 2 linked loci based on recombinations of maternal and paternal *DNA*. Matrices with orthogonality properties, whose elements are either +1 or −1, play a fundamental role in the definition and computation of recombination probabilities. It is also assumed in this formulation that meiosis in a diploid species is regular and balanced so that the phenomenon of gene conversion is not taken into account. At the end of this chapter a paper devoted to understanding the molecular basis of genetic recombination is cited and given special attention. In chapter 3 the recombination probabilities defined in chapter 2 along with a linkage distribution for any number *N* ≥ 2 linked loci are applied in considering linkage and recombination in large random mating diploid populations with no mutation or selection. Briefly, it is shown that in the long run, when there is no mutation or selection, a population will attain a linkage equilibrium with respect to a large number of linked loci with an arbitrary number of alleles at each locus. The results of chapter 3 will useful for investigators interesting in testing for linkage equilibrium in genome wide sweeps for signatures of selection when more than two loci are under consideration.

In chapter 4 the study of evolution with mutation and selection is undertaken within the framework of the two allele Wright–Fisher process that has been frequently used by workers in evolutionary genetics. Unlike the usual analysis of this widely used process that have been based on a diffusion approximation of the process, in chapter 4 the analysis is devoted solely to the applications of matrix algebra to formally study the implications of the assumptions underlying this process along with the assumption that total population size is constant from generation to generation. Among the many formulas derived in this chapter are fixation (absorption) probabilities, the stationary distribution of the case two alleles may mutate among each other and a formula for the quasi-stationary for the case of finite but many absorbing states. Illustrative computer experiments are also reported in this chapter for the case of small populations of about 500 individuals. When population size is large, matrix algebra methods are no longer useful when working out the implications of the assumptions underlying Wright–Fisher process with two or more alleles at some autosomal locus. In chapter 5, Monte Carlo simulation methods are introduced as an alternative theoretical and practical approach to study the implications of assigned numerical values to the parameters of a multitype Wright–Fisher process. The results of a number of computer simulation experiments are reported in this chapter along with a model of inherited autism in human populations which is based on deleterious mutations at many autosomal loci. Some evolutionary implications of the model of inherited autism are also worked out in a Monte Carlo simulation experiment reported in this chapter. With regard to openness with respect to the random number generator used in all the Monte Carlo experiments reported in the book, an outline of the technical account on random number generators giving in Mode and Gallop (2008) was also included in this chapter.

The study of mutations at the *DNA* level begins in chapter 6, where Markov jump processes in continuous time are used to model transitions, nucleotide substitutions, among the four bases *A*, *T*, *C*, *G* that make up a strand of *DNA*. These four bases are viewed as the states of a Markov jump process in continuous time for any base site of a strand of *DNA*. Unlike the Wright–Fisher processes studied in chapters 4 and 5 in which evolutionary time was expressed in terms of generations, the time scale under consideration in these models of nucleotide substitutions is continuous evolutionary time measured in some unit of time such as a year. Among the many examples considered in this chapter is the simple Jukes–Cantor process. In general, for each site of a strand of *DNA*, 12 parameters must be specified to take into account all possible transitions among the 4 bases. Consequently, if one wishes to consider a model of nucleotide substitutions that accommodates a large number of sites of a strand of *DNA*, then number of parameters that must be specified becomes prohibitively large from a practical point of view if one wishes to consider cases in which rates of mutation vary among the sites of a *DNA* molecule.

In order to cope with model that accommodates a large number of sites on a strand of *DNA*, it was decided to formulate a model such that the parameters at each site were viewed as realizations of a Gaussian process that depended on only a few parameters and realizations of this process were mapped into suitable intervals of evolutionary time in which time was positive. The details as to how such a process may be constructed are given in chapter 7 of the book. Chapter 8 contains an account of procedures that may be used to simulate Monte Carlo realizations of the process along with statistical procedures to summarize the simulated data. In this chapter the results of a computer experiment to simulate parallel and back mutations in the *D*-loop of human mitochondrial *DNA* are reported. Briefly, parallel mutations refer to the same mutation at some site in the *D*-loop that may occur in separate linages, which complicates classifying a population into Haplogroups based on human mitochondrial *DNA*. Similarly, back mutations at the same site are also another complicating factor in classifying Haplogroups. In this experiment the number of parallel and back mutations that one may expect during a period of evolution were estimated. At the ends of chapters 6–8 a number of books and papers on Bioinformatics are cited along with an account of the genographic project and a technical paper on problems arising in the classification of haplogroups based on mutations in human mitochondrial *DNA*.

In chapter 9 the ideas underlying the formulation of self regulating branching processes are introduced along with Monte Carlo methods for simulating genealogies and coalescence in a simulated genealogy for the one-type branching process. Coalescence is characterized in terms of the number of generations back such that two randomly selected individuals at some generation in a simulated genealogy have a common ancestor. By selecting a random sample of pairs of individuals it was possible to estimate and plot the distribution of the number of generations back in time for which two randomly chosen individuals have a common ancestor. It is also in this chapter that the ideas of embedding a deterministic model in a self regulating branching process were introduced for both the one and multitype cases. Moreover, in this chapter, the computer experiments using on the one type model are reported in which the estimated quantile trajectories, based on Monte Carlo samples, were compared with the trajectory of the embedded deterministic model. The results of an experiment, in which the embedded deterministic model converges to a period two trajectory are also compared with estimated quantile trajectories of the process with the same numerical assignments to the parameters as those of the deterministic model. Also included in this chapter is the description of a methods designed to extend the one type self regulating branching process to a self regulating multitype model in which conditional probabilities of mutations among the types per generation are included in the formulation.

In chapter 10 a three type self regulating branching process is used in computer simulation experiments to the study the emergence, survival and extinction of mutant types in populations of self replicating individuals evolving from small founder populations. The reasons for choosing to use a three type branching process are twofold. When considering the emergence mutations in such populations, it is of interest to accommodate both deleterious and beneficial mutations in an experiment. Thus, within the framework of a three type process, it was possible to consider experimental scenarios in which a population evolving from an ancestral population, consisting of only of individuals of one single type, that evolves after a few or many generations to contain individuals carrying either beneficial or deleterious, which may arise through the process of mutation among the types that are passed on from generation to generation. There is also another practical reason for considering only three types. For if more than three types are considered in a computer simulation experiment, then it is more difficult statistically to provide informative summarizations of samples of simulated data than if only three types are considered in an experiment. If a reader is interested in other illustrative experiments using the three type model of chapter 10, it is suggested that Mode et al. (2011b) be consulted.

In the models with three types considered in this chapter, selection is partitioned into two components. One component is reproductive success, which is characterized in terms of the expected number of offspring produced by each individual per generation that may differ among types, genotypes, of individuals. The other component of selection is that individuals on some type may be better adapted at utilizing environmental resources and will thus have competitive advantage over individuals in the sense that they are able to survive and produce offspring than types of individuals that are less able to compete for environmental resources. In chapter 10, the results of computer simulation experiments are reported in which if individuals of one genotype contributes on average more offspring to the next generation than other genotypes, then in the long run individuals of this genotype will become predominate in the population. Similarly, if individuals of one genotype are capable to surviving and producing offspring at higher population densities, then individuals of this genotype will eventually become predominate in the population even though the expected number of offspring produced by individuals of all genotypes is the same. Among the references cited at the end of chapter 10 is a book dealing with conceptual issues in evolutionary biology. Among these issues is that of defining of the concept of fitness. It is suggested that the idea of partitioning the evolutionary process of selection into components may be helpful in developing the concept of fitness and its application in theories of evolution.

The theme of partitioning selection into its components is continued in chapter 11, where a two sex model is formulated within the framework of a self regulating branching process in which the genotypes of the females and males are designated. From the genetic point of view, one autosomal locus is under consideration in some diploid species such there are two alleles for each sex that may mutate to the other allele with fixed probabilities per meiosis. In this model it is assumed that selection occurs at the genotypic level, and that in the formation of sexual partnerships sexual selection may occur, because females and males, of each of the three genotypes, may prefer one genotype over the other as sexual partners. Reproductive success is characterized at the partnership level in terms of parameters denoting the expected number of offspring each type of partnership contributes to the next generation. This formulation also includes a component of selection that provides for the possibility that differences among the three genotypes to compete for environmental resources in both sexes may be studied in computer experiments, which are quantified in terms of beta parameters that may differ among genotypes of both sexes with respect to the ability to compete for environmental resources. It is suggested that if a reader is interested in an experiment in which mutations did not emerge in a population that evolved according to the embedded deterministic model but were actually realized among a sample of Monte Carlo realizations of the stochastic process, the paper by Mode et al. (2011a) may be consulted. In chapter 11, the results of several computer simulation experiments with formulation are reported, including one dealing with the evolution of inherited autism in human populations.

In chapter 12, the model formulated in chapter 11 is extended to the case of an age-structured two sex population with overlapping generations. All the components of selection that were included in the model described in chapter 11 are also included in this model, but there is another component of selection the provides for the possibility that each of the genotypes for both sexes may have shorter or longer expected lifespans, which is another aspect of the effects of survivability of individuals in evolution. The computer implementation of an age structured model is much more difficult than the one considered in chapter 11. Consequently, in chapter 12 only the results of experiments using the embedded deterministic model are reported. But, in a revised formulation, a stochastic version of the model has been accomplished and the results of computer experiments with this model have been reported in the paper, Mode et al. (2013), which is now in press and to be published in 2013.

Chapter 13 is devoted to a review of the concept of a gene with a view toward the development of methods for simulating the evolution regions of a genome that contain genes. After a review of the literature on the changing concept of a gene, an updated working definition of a gene is given based on recent experiments using micro chip technology in which the products of transcription were studied. This definition includes the phenomenon of alternate splicing of three letter codons as well as *RNAs* that are not involved in the coding of proteins but may have some regulatory function. Because of difficulties that my arise when gene regulation is taken into account, this definition does not include regions of *DNA* in a genome that may be involved in the regulation of genes. However, to provide some insights into the regulation of genes, two examples from human blood groups and the *Shh* locus in mice are described which include not only regions of a gene that code for proteins but also genomic regions involved in the regulation of these genes. Listed at the end of this chapter are about 70 references on applications of statistical methods, methods published in journals of Bioinformatics as well as some from classical genetics that were cited in connections with developing a recent working definition of a gene.

The last chapter of the book that is devoted to substantive concepts is chapter 14 in which statistical problems of detecting genomic signals of selection are reviewed and described along with a review of published methods for simulation regions of a genome. Whenever the mathematical basis underlying this methods was judged to be inadequately described, the author or authors were criticized for lack of transparency with a suggestion that it would be advisable if individuals with professional training in mathematics were also included on the team to increase the level of transparency. New algorithms for simulating such phenomena as gene conversion, genetic recombination as well as types of mutations including nucleotide substitutions, inversions, deletions and copy number variation are described in this chapter. But, as yet, only a few preliminary computer experiments have been conducted as proofs of concepts to show that the development of such methods is indeed possible. It is suggested that the contents of chapter 14 are part of the quest by many investigators to work toward the grand challenge of evolutionary and population genetics as outlined by Akey and Shriver (2011). At the end of this chapter 50 references are cited consisting mostly of literature on statistical and other methods from Bioinformatics that have been used by many investigators in the development of the contents of chapter 14. Finally, the book ends in chapter 15 with suggestions for future research and further readings on evolution and its applications for human societies at large.

## 5. An example in which the predictions of embedded determinist model are not consistent with those of the stochastic process

In this section the results of a simulation experiment will be reported in which the driving force of evolution by natural selection was a reproductive advantage of a mutant genotype. To quantify the idea of a reproductive advantage for mutant genotype 3, the vectors of lambda parameters were assigned the values

(5.1) λ=(1.05, 1.05, 1.5),

indicting on average that genotype 3 produced an average of 1.5 offspring per generation; whereas genotypes 1 and 2 produced an average of 1.05 offspring per generation. For this experiment, the values of the conditional probabilities in the matrix M in Equation (3.1) were assigned the values shown in the matrix

(5.2) $$M=\left(\begin{array}{ccc} \mu_{11} & 10^{-6} & \;0\\ 10^{-12} & \;\mu_{22} & 10^{-14}\\ 10^{-15} & 10^{-17} & \;\mu_{33} \end{array}\right).$$

Note that the values on the principal diagonal of the matrix were determined such that the sum of each row in the matrix was 1.

As shown in section 3, in the multitype self regulating branching process with three types under consideration, Weibull type survival depending on two parameters was used. To simulate neutral evolution, for each genotype the two parameters in the survival function were assigned the same values. Thus, in all the experiments reported in this paper, the alpha parameters were assumed to be equal and were assigned the value α_*i*_ = 2 for each genotype *i* = 1, 2, 3. Similarly, the beta parameters, which govern the ultimate size of the population for each genotype, were assigned the values α_*i*_ = 2 for each genotype *i* = 1, 2, 3. Similarly, the beta parameters, which govern the ultimate size of the population for each genotype, were assigned the values β_*i*_ = 10^−6^ for each genotype *i* = 1, 2, 3. The rationale for assigning these values was to conduct a computer simulation experiment in which a rare mutation may occur, but the size of the population for each genotype was restricted to about 10^6^ individuals. Finally, it was assumed that the initial vector for a process evolving under the above assumptions was

(5.3) X(0)=(10, 000, 0, 0),

indicating that the initial population was composed only of 10,000 individuals of genotype 1 so that as the simulated population evolved genotypes 2 and 3 could only arise as a result of the process of mutation.

At the outset of this experiment it was known that no individuals of mutant genotype 3 would appear in the simulated population using Monte Carlo methods to simulate 100 replications of 6000 generations of evolution. The reason, underlying the justification of this statement, was that for every computer simulation experiment, using Monte Carlo simulation methods, the seed for the random number generator, an initial assigned number to the random number generator used in the sequential calculation of the random numbers used in any experiment, was, as a matter of policy, the same in every experiment. An advantage of using this technique is that it is possible to duplicate any Monte Carlo simulation experiment, if, for any reason, such as a loss of power, the results of any experiment are lost, it be possible to repeat the experiment using the same set of computer generated random numbers. It seems paradoxical that even if a sequence of numbers is computed by a recursive deterministic procedure, the resulting sequence of numbers often pass statistical tests for randomness. Consequently, such numbers may be used in Monte Carlo simulation experiments with confidence. If a reader is interested in more details regarding the calculation of random numbers, the paper by Mode and Gallop (2008) and the references cited therein may be consulted.

**Figure 1:**
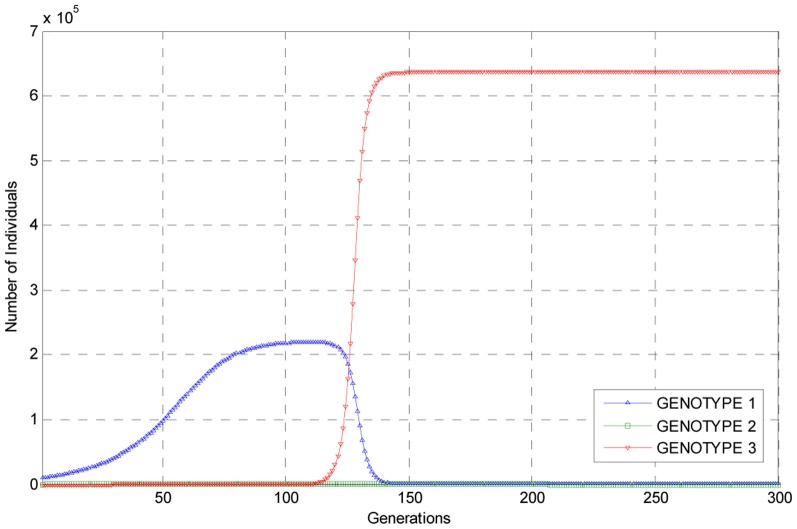
**Graphs of the trajectories of the numbers of each of the three genotypes as estimated using the embedded deterministic model**.

Presented in Figure 1 are graphs of the estimated number of individuals of each genotype for the first 300 generations of the experiment as predicted by the embedded deterministic model.

As can be seen from these trajectories, the estimated number of individuals of genotype 1 rises to a level of about 2 × 10^5^ individuals at about 120 generations into the experiment, and then the population of individuals of this genotype undergoes a steep decline so that the number of individuals of this genotype are small when compared to the number of individuals of genotypes 3, which, by assumption, had a reproductive advantage. As the number of individuals of genotype 1 in the population declined, the numbers of individuals of mutant genotype 3 rose to over 6 × 10^5^ by generation 150 and thus become predominant in the population. The number of individuals of genotype 2 remained relatively small throughout the experiment as can be seen from the trajectory for this genotype. But it is clear from the trajectories plotted in Figure 1 that, according to the embedded deterministic model, that individuals of genotype 3 did become predominant in the population during the first 300 generations of simulated evolution. It is also interesting to note that within 300 generations, each of the three trajectories corresponding to the three genotypes has converged to constants, indicating this is this experiment the population evolved rather rapidly.

**Figure 2:**
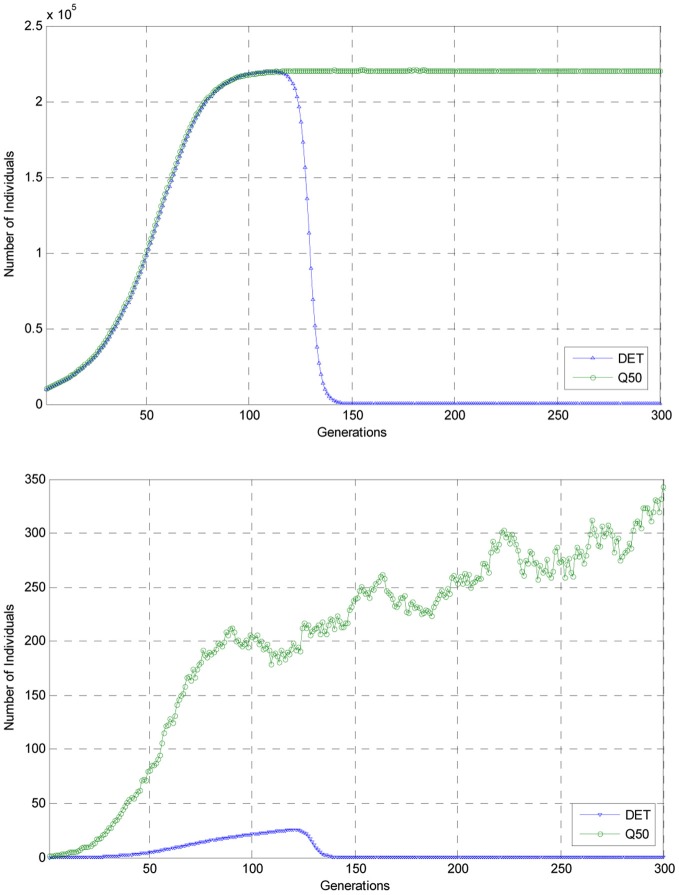
**Comparisons of the deterministic trajectories of genotypes 1 and 2 with the *Q*50 trajectories for the first 300 generations of the experiment**.

Presented in Figure 2 in the upper panel are graphs of the trajectory for genotype 1 as computed using the embedded deterministic model as well as the *Q*50 trajectory for genotype 1 as estimated from a sample of 100 Monte Carlo replications of 6000 generations of evolution. In the lower panel in this figure, these same trajectories for the number of individuals of mutant genotype 2 are also plotted for the first 300 generations of the experiment.

As can be seen from the deterministic trajectory, *DET*, and the estimated *Q*50 trajectory of the stochastic process for genotype 1, as shown in the upper panel, by generation 150 the *Q*50 trajectory had reached a nearly constant level of over 2 × 10^5^ individuals and remained at this level for the remaining generations of evolution shown in this figure. This nearly constant level indicated the among the 100 realizations of the stochastic process for the first 300 generations of simulated evolution, genotype 3 did not appear the population. Indeed from a more detailed inspection of the simulated data for 6000 generations of evolution replicated 100 times, it was observed that mutant genotype 3 did not appear in the population. But, as shown in this figure and also Figure 1, according to the predictions of the embedded deterministic model, the number of individuals of genotype 1 had declined to a small number when compared to that predicted by the stochastic process and mutant genotype 3 had risen to predominance in the population. Thus, in this illustrative example, it is clear that the embedded deterministic model failed to predict the evolution of the stochastic process in the sense that *DET* differed significantly from the estimated *Q*50 trajectory of the process for this genotype.

Shown in the lower panel of Figure 2 are the trajectories *DET* and *Q*50 for the embedded deterministic model and the stochastic process for genotype 2. When, as shown in this lower panel, the vertical axis of the graph depicts populations sizes from 0 to 350, the graphs of the trajectories *DET* and *Q*50 are more clearly differentiated. For in this lower figure, it can be seen that throughout the first 300 generations of simulated evolution, the deterministic trajectory, *DET*, for individuals of genotype 2 remained below 50 individuals, but that for the *Q50* of the stochastic process had reached nearly 350 individuals by generation 300. In this experiment, there was also apparent that the trajectory computed using the embedded deterministic model was not a good predictor of the *Q*50 trajectory of the stochastic process for individuals of genotype 2. Evidently, among the 100 realizations of simulated evolution of the stochastic process for 6000 generations of evolution, the number of individuals of genotype 2 never rose to a number sufficiently large to ensure that the mutation *A*_2_ → *A*_3_ occurred with positive probability. This experiment also suggests that if the carrying capacity of the environment is such that a large populations of individuals cannot not supported, then it is more unlikely that rare beneficial mutation will appear in an evolving population due to insufficient total population size.

It is known from experiments not reported here that by increasing the probability of the mutation *A*_2_ → *A*_3_, mutant genotype 3 would appear in the population and become predominant in a Monte Carlo simulation experiment. It is also known from experiments reported in chapter 10 of the book Mode and Sleeman (2012) that if one assumes that individuals of genotype 3 have a selective advantage over the other two genotypes by assigning a smaller value of the beta parameter for genotype 3, then eventually, even if selection is neutral with respect to reproductive success, individuals of genotype 3 will become predominant in the population. While carrying out the computer experiments reported in the book, it was not observed that a rare beneficial mutation would fail appear in the population, using the self regulating process three type branching process under consideration. But if a reader is interested in experiments using this model such that an advantageous mutant genotype did not appear in the population in experiment completed after work on the book was finished, the recent paper of Mode et al. (2011b) may also be consulted, where other experiments in which the predictions of the embedded deterministic model are not consistent with those of the process.

Many models used in evolutionary and population genetics in the past, as well as models used optimize the process selection in domestic livestock, have been based on the premise that the population is of infinite size, and, therefore, the use of deterministic models was justified. It has sometimes been stated that even though a stochastic model should be used, the trajectory of a deterministic model is a measure of central tendency for the stochastic process. But, actually, wild and natural population are finite, especially in populations of domestic livestock and in natural population at the beginning of their evolution or in bottlenecks, such that for some reason, total population size has been reduced to a small number. In this connection, in an interesting paper by Costard and Elsen (2011) on the optimization of gene-assisted selection in small populations of domestic livestock, the deterministic and stochastic approaches were compared. These authors concluded that when selection involves one inherited gene, the finite or stochastic case, should be considered. And, as shown in an example in this paper as well as in other published work with another model, see Mode et al. (2011a), it is relatively easy to construct examples in which the embedded deterministic model fails to predict the results observed in a Monte Carlo implementation of the stochastic process, particularly in connection with the occurrence of beneficial mutations.

As the software needed to implement stochastic processes become more readily available with user friendly front ends to expedite their applications to problems of simulation the evolution of natural or domestic populations of plants and animals, the sometimes onerous task of writing and debugging software will be relegated to those specializing in software development and an experimenters will be free to apply the software as an aid to finding solutions to the conceptional evolutionary problems that confront them. As the use of inexpensive desk top computers with high speeds of execution of computation and large memories become widely available, the time taken to complete Monte Carlo simulation experiments will be reduced to shorter time periods that will, in principle, encourage their use by increasing numbers of researchers.

### Conflict of interest statement

The authors declare that the research was conducted in the absence of any commercial or financial relationships that could be construed as a potential conflict of interest.
